# The Clinical and Molecular Characteristics of Systemic Mastocytosis with Associated Non-Mast Cell Myeloid Neoplasm

**DOI:** 10.3390/jcm15187232

**Published:** 2026-09-17

**Authors:** Neha Seth, Pratik Q. Deb

**Affiliations:** 1 Department of Pathology and Laboratory Medicine, Northwell Health, New Hyde Park, NY 11042, USA; nseth1@northwell.edu; 2 Department of Pathology and Laboratory Medicine, Donald and Barbara Zucker School of Medicine at Hofstra/Northwell Health, Hempstead, NY 11549, USA

**Keywords:** mastocytosis, systemic mastocytosis, systemic mastocytosis with associated hematologic neoplasm, systemic mastocytosis with associated myeloid neoplasm, myeloid neoplasm, mast cell leukemia

## Abstract

**Background:** Systemic mastocytosis (SM) with associated non-mast cell myeloid neoplasm is a rare condition, defined by the presence of both SM and a clonal non-mast cell neoplasm. Due to its rarity, the demographic and molecular characteristics are poorly understood. This study investigated seven patients diagnosed with this condition at our institution. **Methods:** Our institutional database was searched for “systemic mastocytosis” from 1 January 2020, to 31 December 2025. Each case was annotated to identify the primary cohort of this study, systemic mastocytosis with associated non-mast cell myeloid neoplasm (SM-AHN). Demographic characteristics, outcome, hematologic features at diagnosis, cytogenetics, and molecular characteristics were annotated. Eight cases of isolated SM diagnosed during the same period were also analyzed for comparison. All patients in the database were followed until their death or until 31 December 2025. All data were exported to SPSS v. 28 (IBM, Armonk, NY, USA^®^). **Results:** A total of seven patients were diagnosed with SM-AHN. Compared to isolated SM, SM-AHN patients showed older age at presentation, poorer hematologic values (increased anemia and thrombocytopenia), higher mutational burden, and poorer outcomes. *KIT* mutations were most common, predominantly D816V, with other variants like D816Y and L576F also observed. Additional frequent mutations included *DNMT3A*, *TET2*, *ASXL1*, *SF3B1*, *EZH2*, *JAK2*, *NRAS*, and *SRSF2*. **Conclusions:** Our findings, though limited by a small sample size, suggest that SM with associated myeloid neoplasm presents with distinct clinical and molecular features compared to isolated SM. This combined disorder is associated with older age, worse hematologic parameters, a higher mutational burden, and poorer prognosis. The frequent co-occurrence of mutations in other genes, in addition to KIT mutations, highlights the complex molecular landscape.

## 1. Introduction

Systemic mastocytosis (SM) is a clonal hematopoietic neoplasm characterized by the pathological accumulation of abnormal mast cells (MCs) in extracutaneous organs: most commonly the bone marrow, skin, gastrointestinal tract, liver, and spleen [1,2]. It is typically driven by activating mutations in the *KIT* gene, particularly D816V, which lead to constitutive activation of the KIT receptor tyrosine kinase and uncontrolled MC proliferation [3]. SM’s clinical presentation is highly variable, ranging from indolent disease with minimal symptoms to aggressive forms with organ dysfunction [4].

The disease encompasses a spectrum of variants: indolent SM, smoldering SM, aggressive SM, SM with an associated hematologic neoplasm (SM-AHN), and mast cell leukemia (MCL). Both the 2022 World Health Organization (WHO) classification and the 2022 International Consensus Classification (ICC) delineate these subtypes, with minor differences in nomenclature and diagnostic criteria [5,6]. Notably, while the WHO uses the term “associated hematologic neoplasm” (AHN), the ICC uses “associated myeloid neoplasm” (AMN) to highlight that most of these independent neoplasms are of myeloid origin. Both classifications rely on a combination of major and minor criteria, including histologic evidence of multifocal dense MC aggregates, aberrant MC morphology and immunophenotype, activating *KIT* mutations, and persistently elevated serum tryptase levels. In this manuscript, hereon, we have used the term SM-AHN.

SM-AHN is defined by the coexistence of SM and an independent clonal hematologic neoplasm, typically a myeloid malignancy such as myelodysplastic syndrome (MDS), myeloproliferative neoplasm (MPN), chronic myelomonocytic leukemia (CMML), or acute myeloid leukemia (AML) [7,8]. However, rare cases involving chronic lymphocytic leukemia (CLL) and chronic myeloid leukemia (CML) have also been reported [9,10]. SM-AHN is an advanced form of SM associated with a worse prognosis than isolated SM [11,12]. Diagnosis requires independently fulfilling the criteria for both SM and the associated neoplasm. Clinically recognizing SM-AHN is crucial, as management must address both components to avoid suboptimal treatment and poor outcomes. By detailing key diagnostic indicators such as specific demographics, severe cytopenias, and complex mutations our study equips oncologists and pathologists to accurately identify both disease components, which is critical for guiding effective dual-targeted therapies and improving patient outcomes.

The molecular landscape of SM-AHN is complex, often involving additional mutations in genes such as *DNMT3A*, *TET2*, *ASXL1*, *SF3B1*, *EZH2*, and *SRSF2* alongside *KIT* mutations [13]. These mutations influence prognosis and guide therapy, particularly given the emergence of targeted agents like midostaurin and avapritinib for *KIT*-mutated SM [14,15].

Despite advances in classification and molecular diagnostics, the demographic features, hematologic parameters, molecular characteristics, and outcomes of SM-AHN remain undercharacterized, particularly in single-institution cohorts. This study addresses this gap by analyzing a series of SM and SM-AHN cases from our institution, focusing on demographics, laboratory findings, molecular profiles, and survival outcomes.

## 2. Materials and Methods

### 2.1. Patient Selection and Data Collection

We retrospectively searched our institutional database for pathology reports containing the term “systemic mastocytosis” between 1 January 2020, and 31 December 2025. Two hematopathologists independently reviewed all retrieved cases, explicitly excluding cutaneous mastocytosis to restrict the cohort to SM. Included cases were then classified as isolated SM or SM-AHN according to the WHO 2022 and ICC 2022 criteria. Demographic data (age at diagnosis, sex, diagnosis date, last follow-up date, and final status), hematologic parameters (hemoglobin, platelet count, white blood cell count, and peripheral blood blast percentage), and serum lactate dehydrogenase (LDH) levels were extracted from electronic medical records.

### 2.2. Hematologic and Morphologic Analysis

Complete blood counts, peripheral blood smears, bone marrow (BM) aspirates, and BM core biopsies were reviewed. Our routine BM immunohistochemistry (IHC) panel (CD34, CD117, CD71, E-cadherin, MPO, CD15, CD61, and factor VIII) is designed to identify and classify myeloid neoplasms. Within this panel, CD117 highlights MCs due to their strong expression. Additional MC-specific markers (CD2, CD25, CD30, and tryptase) were incorporated for further classification.

### 2.3. Cytogenetic and FISH Analysis

Conventional karyotyping and fluorescence in situ hybridization (FISH) were performed for all cases as part of the standard diagnostic evaluation.

### 2.4. Molecular Studies

Targeted next-generation sequencing (NGS) was performed using the OnkoSight myeloid panel (BioReference Health™, Elmwood Park, NJ, USA), which evaluates up to 50 genes, including: *ABL1*, *ASXL1*, *BCOR*, *BCORL1*, *BRAF*, *CALR*, *CBL*, *CDKN2A*, *CSF3R*, *DNMT3A*, *ETV6*, *EZH2*, *FBXW7*, *FLT3*, *GATA2*, *HRAS*, *IDH1*, *IDH2*, *JAK2*, *KIT*, *KRAS*, *MPL*, *MYD88*, *NPM1*, *NRAS*, *PHF6*, *PTEN*, *PTPN11*, *RUNX1*, *SETBP1*, *SF3B1*, *SRSF2*, *TET2*, *TP53*, *U2AF1*, *WT1*, and *ZRSR2*.

### 2.5. Droplet Digital PCR Analysis

Droplet digital PCR (ddPCR) was performed on peripheral blood samples from all patients concurrently at the time of diagnosis to enable highly sensitive detection and quantitation of targeting *KIT* D816V at the limit of detection of 0.03% variant allele frequency. Non D816V mutations were not targeted by ddPCR.

### 2.6. Statistical Analysis

Descriptive statistics were used to summarize demographic and laboratory data. Kaplan-Meier survival analyses were performed using SPSS v28 (IBM) to compare overall survival between the isolated SM and SM-AHN groups. Heatmaps were generated to visualize mutational profiles. Log rank test was performed for comparison of survival between two groups.

## 3. Results

### 3.1. Overview of Cases

Our cohort included 15 cases of SM diagnosed between January 2020 and December 2025. Seven cases were classified as SM-AHN and eight as isolated SM. Detailed clinical, hematologic, and molecular features are summarized in Table 1.

### 3.2. Peripheral Blood Findings and Other Laboratory Features

Patients with SM-AHN presented at an older median age (74 years; range, 35–93) compared to those with isolated SM (54 years; range, 32–76). SM-AHN patients exhibited more pronounced cytopenias, including a lower median hemoglobin (8.3 g/dL vs. 13.2 g/dL) and frequent thrombocytopenia (median platelets 58 × 10^3^/µL vs. 239 × 10^3^/µL). Although average WBC count was higher in SM-AHN group (19 × 10^3^/µL), it was driven by outliers as the median WBC count is comparable to that of isolated SM patients (median of 7.1 × 10^3^/µL vs. 6.3 × 10^3^/µL). Elevated LDH levels (more than 240 IU/L in adults in our laboratory), indicative of increased cell turnover, were predominantly seen in SM-AHN patients.

### 3.3. Bone Marrow Histopathology

Bone marrow examinations of SM-AHN patients revealed variable cellularity ranging from 40% to 90%, with prominent clusters and interstitial infiltration of MCs showing spindled to oval morphology and strong CD117 positivity. Aberrant immunophenotypic expression of CD25 was frequent; however, it was dim or variable in some cases and entirely absent in the MCL patient, while CD2 and CD30 expressions also varied. CD30 positivity was observed in select cases such as SM + MDS-EB1. Associated hematopoietic neoplasms demonstrated characteristic features: SM + AML showed extensive blast proliferation (up to 85% on aspirate), often with eosinophilia and dysplasia; SM + CMML revealed marked monocytosis, dysplastic granulopoiesis, and increased immature monocytes; SM + MDS-LB-SF3B1 and SM + MDS/MPN-RS-T exhibited dyserythropoiesis, ring sideroblasts, and occasional megakaryocytic abnormalities; and SM + MDS-EB1 and MCL cases showed significant reticulin fibrosis (grade 2–3), with diffuse MC infiltration sometimes exceeding 20% of marrow cellularity.

**Figure 1:**
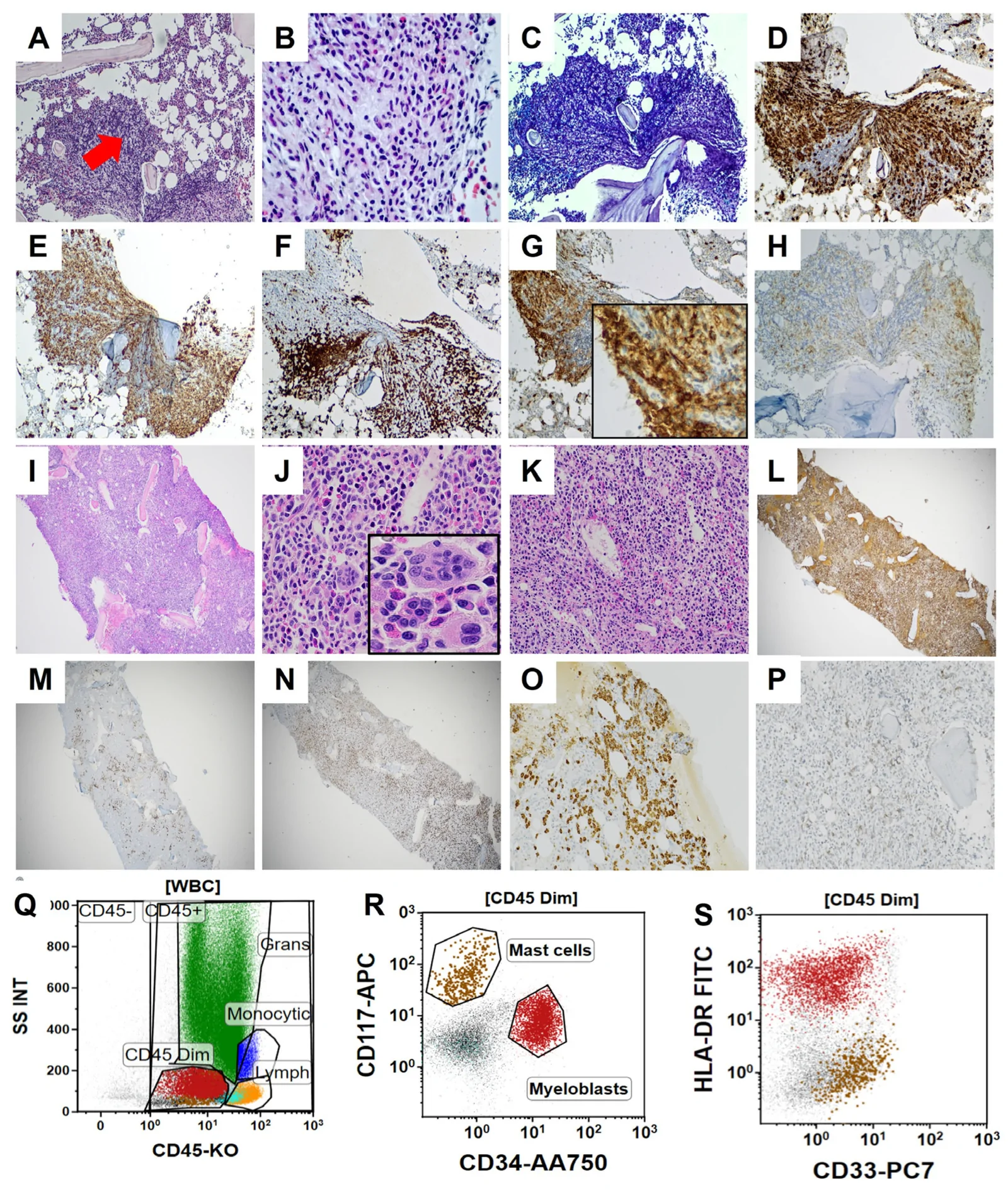
Histomorphological and immunohistochemical features of isolated SM and SM-AHN. (**A**–**H**) Photomicrographs showing features of the bone marrow of a patient with isolated systemic mastocytosis. (**A**,**B**) Photomicrograph showing bone marrow with patchy areas of hypercellularity with accumulated mast cells showing spindling (H&E, 100×, 400×, red arrow showing neoplastic spindled mast cells forming aggregates). (**C**) Giemsa highlights mast cells and their metachromatic granules (Giemsa, 100×). (**D**) CD117 and (**E**) Tryptase highlight mast cells (IHC, 100×) (**F**) CD2 highlights T-cells associated with the mast cell aggregate, but do not highlight mast cells. Mast cells show aberrant expression of (**G**) CD25 and (**H**) CD30 (variable). (**I**–**P**) Photomicrographs showing features of the bone marrow of a patient with SM and MDS/MPN-RS-T (**I**–**K**) Photomicrograph of a bone marrow biopsy showing markedly hypercellular marrow with myeloid predominance and megakaryocytic atypia (H&E, 40×, 100×, inset 400×). (**L**) Myeloperoxidase and (**M**) CD71 show the myeloid predominance of the marrow (IHC, 40×). (**N**) CD117 (IHC, 40×) and (**O**) Tryptase (IHC, 100×) highlight mast cells. Mast cells show aberrant expression of (**P**) CD25 (IHC, 100×). (**Q**–**S**) Flow cytometric features of the bone marrow aspirate of an SM-AHN patient. Showing mast cells and myeloblasts present in the dim CD45 gate with mast cells showing characteristic bright CD117 and lack of HLA-DR expression.

The histomorphological and immunophenotypic findings from the bone marrow biopsies of representative cases are depicted in Figure 1.

### 3.4. Cytogenetic and Molecular Features

Cytogenetic analyses revealed diverse abnormalities in the SM-AHN group, including inv(16) in SM + AML, trisomy 21 mosaicism in SM + CMML, del(9q) in SM + MDS-EB1, and loss of Y in SM + MDS/MPN-RS-T. Remaining cases exhibited normal karyotypes. FISH confirmed relevant rearrangements, such as CBFB in SM + AML.

Next-generation sequencing identified frequent *KIT* mutations (primarily D816V, occasionally D816Y) across all SM-AHN cases. These were often accompanied by high-variant allele frequency (VAF) mutations in *ASXL1*, *TET2*, *DNMT3A*, *SF3B1*, *U2AF1*, *JAK2*, *NRAS*, and *RUNX1*, suggesting clonal complexity. Conversely, isolated SM cases primarily displayed *KIT* D816V with few additional mutations and lacked significant cytogenetic abnormalities. The status of *KIT* D816V (positive vs. negative), detected by NGS on bone marrow, was concordant with its detection status on peripheral blood by ddPCR.

**Figure 2:**
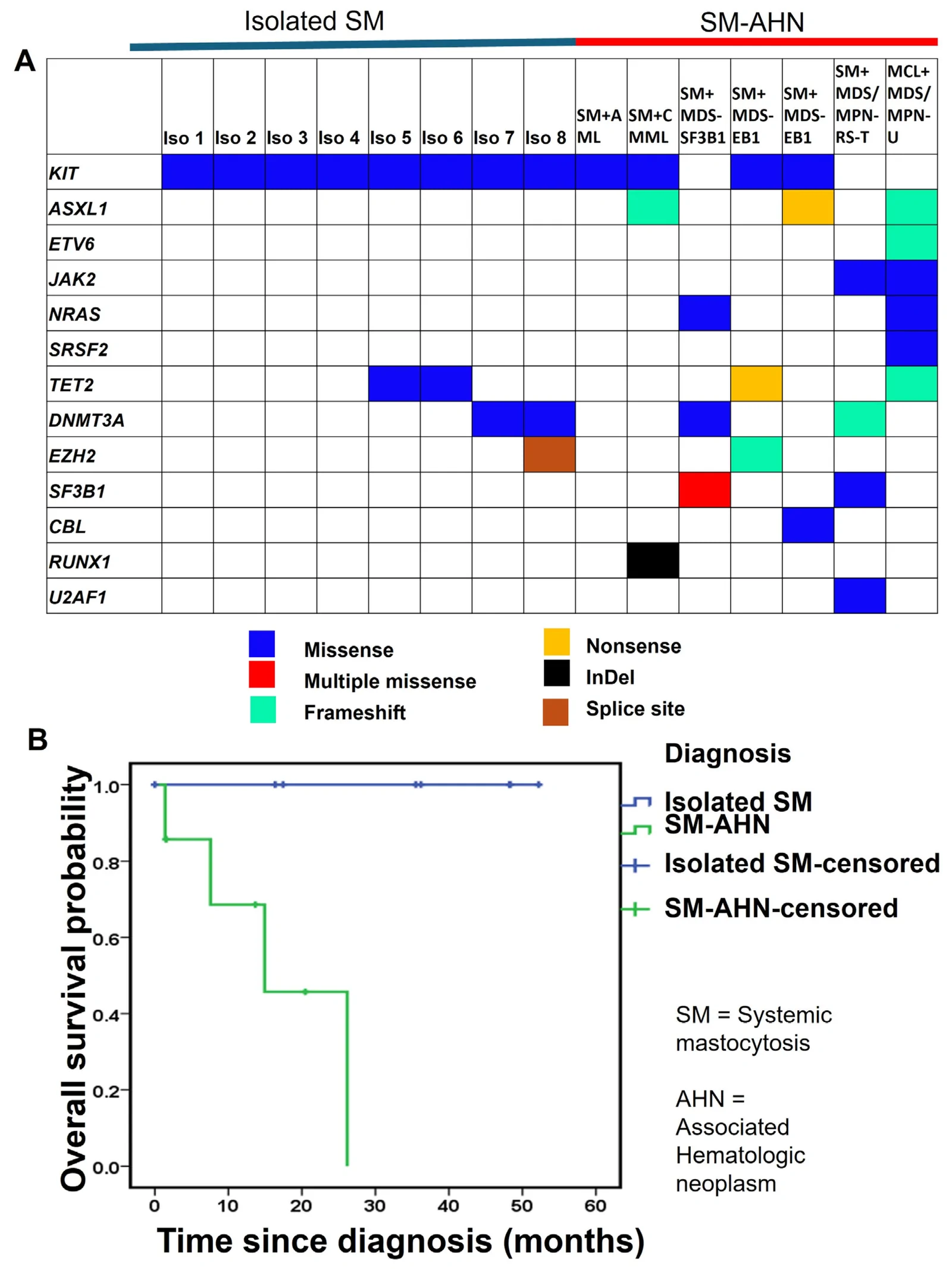
(**A**) Molecular features of isolated SM and SM-AHN showing higher frequency of genetic alteration in SM-AHN patients. (**B**) Comparative survival outcome of SM and SM-AHN patients.

The molecular features are depicted in Figure 2A.

### 3.5. Clinical Outcome

Over a median follow-up of 31 months, mortality events occurred in four out of seven SM-AHN patients, compared to none out of eight isolated SM patients. Kaplan-Meier survival analysis demonstrated significantly poorer overall survival in patients with SM-AHN (median 26 months) compared to those with isolated SM (median not reached; log rank test *p* = 0.001). (Figure 2B). Because one group (isolated SM) shows zero event, hazard ratio and 95% confidence interval couldn’t be reliably calculated.

## 4. Discussion

Our study provides an in-depth look into the clinical, morphologic, and molecular landscape of SM-AHN, a challenging entity that is often underrecognized in clinical practice. Our cohort highlighting the demographic and laboratory differences between SM-AHN and isolated SM, reinforces the importance of integrating multiple diagnostic modalities, and highlights the need for heightened clinical awareness to improve outcomes in this complex disease.

This study depicts that SM-AHN is a clinically and molecularly distinct entity from isolated SM, characterized by an older age at presentation, more severe cytopenias, a higher mutational burden, and significantly poorer prognosis. Our findings underscore a critical diagnostic pitfall: the potential for the associated myeloid neoplasm to morphologically overshadow the mast cell component, leading to incomplete or delayed diagnosis. A key challenge in diagnosing SM-AHN is that the bone marrow is often dominated by features of the associated neoplasm, such as extensive myeloblasts in AML or marked monocytosis in CMML, which can mask the more subtle mast cell infiltrate. Although the SM and myeloid components in our cohort were diagnosed simultaneously through upfront multi-modal testing, in broader clinical practice, the unexpected discovery of a *KIT* mutation on a myeloid NGS panel frequently serves as the critical trigger prompting pathologists to re-evaluate a biopsy for masked SM. This makes a high index of suspicion and a comprehensive diagnostic approach essential. Routine Giemsa staining, performed in our laboratory for all bone marrow biopsy evaluation, might be one of the reasons for us not missing any subtle and masked mast cell aggregates in SM-AHN cases. Hence, adaptation of this inexpensive technique might be considered.

Our experience confirms the indispensability of specific ancillary tests. Giemsa staining, which vividly highlights metachromatic mast cell granules, serves as an excellent and cost-effective screening tool. Diagnosis should be confirmed with a targeted immunohistochemical panel, where aberrant co-expression of CD25 on CD117-positive mast cells is a key indicator of neoplasia [16,17,18]. CD2 and CD30 can also be helpful, particularly in more advanced cases, though their expression may be variable [19,20]. Additionally, flow cytometry can identify neoplastic mast cells by their characteristic bright CD117 and dim CD45 expression, even when they are a minor population [21,22,23]. Even in cases where the mast cell component is overshadowed by a dominant myeloid neoplasm, a careful review of bright CD117-positive populations can reveal subtle mast cell clusters that might otherwise be missed, prompting further evaluation with immunohistochemistry and molecular studies [23,24]. The diagnosis of SM-AHN requires careful navigation of cases with overlapping phenotypes. For example, in patients with core-binding factor AML (e.g., inv(16)), leukemic blasts can exhibit mast cell differentiation, mimicking SM. In our 35-year-old SM + AML patient, this differential was resolved by the presence of dense mast cell aggregates, aberrant CD25 expression, and a *KIT* D816Y mutation—features indicative of a true concurrent SM clone rather than leukemic differentiation. Additionally, our cohort included a mast cell leukemia (MCL) case that lacked both *KIT* mutations and CD25 expression. This diagnosis was firmly established by the presence of diffuse infiltration comprising 20–30% atypical mast cells in the highly fibrotic bone marrow. It is well-documented that aggressive variants like MCL can lack classical *KIT* codon 816 mutations and aberrant CD25 expression, necessitating a strong reliance on core morphological criteria and a comprehensive clinicopathologic synthesis for accurate diagnosis [25].

A key finding from our study is the older median age of SM-AHN patients compared to isolated SM (74 vs. 54 years). This aligns with existing literature suggesting that SM-AHN often emerges as a second hematologic event in older adults, possibly reflecting cumulative mutational burden and clonal evolution [26,27]. The pronounced cytopenias observed in SM-AHN cases, particularly anemia and thrombocytopenia, reflect the additive impact of the associated myeloid neoplasm on marrow function and are consistent with prior studies indicating that cytopenias are a hallmark of advanced mastocytosis subtypes [28]. The genetic landscape of SM-AHN is notably more complex than that of isolated SM. While some isolated SM cases in our cohort did exhibit additional mutations (e.g., *TET2*, *DNMT3A*), these were carefully evaluated against concurrent clinical and morphological criteria. Most additional genetic alterations (other than *KIT*), found in isolated SM occurred at low variant allele frequencies (4% VAF or less). One patient with isolated SM showed *TET2* alteration with high (51%) VAF, likely a germline variant. In the absence of diagnostic morphological dysplasia or overt myeloid proliferation, these additional alterations were classified as clonal hematopoiesis of indeterminate potential (CHIP) or clonal cytopenia of undetermined significance (CCUS), rather than evidence of an associated myeloid neoplasm. SM-AHN cases in our cohort frequently harbored additional high-variant-allele-frequency mutations in genes integral to myeloid neoplasia, such as *ASXL1*, *TET2*, *SF3B1*, and *RUNX1*. The presence of these co-mutations, including disruptive frameshift and nonsense alterations, points to greater genetic instability and likely contributes to the aggressive clinical course [29].

The clinical and mutational profile of our cohort aligns closely with findings from larger, multi-center SM-AHN registries. For example, large cohorts from the Mayo Clinic and the European Competence Network on Mastocytosis (ECNM) consistently report a median age of over 70 years for SM-AHN patients, with CMML and MDS being the most frequently associated myeloid neoplasms [7,27,29]. Furthermore, our observation of a highly complex mutational landscape—specifically the frequent co-occurrence of *ASXL1*, *TET2*, and *SRSF2* alongside *KIT* D816V—mirrors these larger genomic studies, which have established that this multi-mutated profile drives the aggressive phenotype and inferior survival typical of SM-AHN compared to isolated SM [29]. Importantly, the recent ECNM data establishes that the reduced overall survival in SM-AHN is independently driven by the aggressiveness of the mastocytosis component itself. Consequently, our single-institution observations accurately reflect broader, real-world epidemiological trends. This robust concordance emphasizes that precise classification of both disease components is essential for deploying modern, dual-targeted therapies effectively [30].

The high susceptibility of SM to co-evolve with myeloid neoplasms is rooted in the biology of early clonal hematopoiesis. Evidence suggests that some SM-AHN likely originates from a shared multipotent hematopoietic stem/progenitor cell whereas other SM-AHN are combinations of two distinct clonal entity [31]. In most cases, mutations in epigenetic or splicing genes (e.g., *TET2*, *ASXL1*, *SRSF2*) occur as primary, early events leading to a clonal myeloid proliferation, whereas the *KIT* D816V mutation typically arises as a secondary genetic ‘hit’ within this pre-established clone [31]. This shared ontogeny explains why SM is disproportionately associated with specific myeloid neoplasms, such as chronic myelomonocytic leukemia (CMML) and myelodysplastic/myeloproliferative neoplasms (MDS/MPN), which share similar founding mutations [31,32].

These additional mutations are not merely bystanders; they are established as adverse prognostic markers in myeloid malignancies and their presence in SM-AHN underscores its aggressive biology and helps explain the poorer outcomes observed. Among various genetic alterations, mutations involving *ASXL1*, *RUNX1*, and *NRAS* have been proposed to be poorer prognostic markers for advanced SM and were incorporated in the prognostic scoring systems developed by Mayo clinic [33,34]. However, please note that this scoring system was applied to isolated SM only and not reflective of SM-AHN.

The clinical implications of these findings are profound. The median overall survival in our SM-AHN group was only 14 months, contrasting sharply with the unreached median survival in isolated SM, a finding consistent with larger studies where prognosis is primarily driven by the associated neoplasm [7,27]. This highlights the necessity of accurate and complete diagnosis. Failure to identify the SM component can lead to suboptimal treatment, as optimal management of SM-AHN often requires a dual-pronged strategy: targeting the mast cell clone with KIT inhibitors (e.g., avapritinib) while simultaneously treating the associated myeloid neoplasm with appropriate therapy, such as hypomethylating agents or induction chemotherapy [15,35].

This study has limitations inherent to its retrospective, single-center design, and small sample size, which restrict the generalizability of our findings. For instance, our isolated SM cohort consisted of indolent SM, leading to a 100% utilization rate of avapritinib. This uniform reliance on targeted KIT inhibition is not representative of the broader isolated SM population, wherein many indolent cases are managed conservatively with non-targeted, symptom-directed therapies. This reflects a selection bias typical of specialized tertiary care centers. Moreover, our database search strategy—relying on the term “mastocytosis” in pathology reports—introduces a selection bias, as it inherently excludes SM-AHN cases where the subtle mast cell component was entirely overlooked by the initial pathologist and thus never documented. Variations in diagnostic approaches over the study period and limited follow-up in some cases may also introduce bias. Furthermore, fluorescence-activated cell sorting (FACS) of the myeloid and mast cell components followed by separate next-generation sequencing (NGS) was not performed, limiting our ability to definitively assign specific mutations to distinct clonal populations. The approximately 20-year difference in median age between our isolated SM and SM-AHN cohorts serves as a major, unadjustable confounder for overall survival, meaning the poorer prognosis in the SM-AHN group is likely driven by a combination of disease biology and advanced age. Despite these constraints, this analysis provides valuable real-world insights into the diagnostic challenges and complex biology of SM-AHN, emphasizing the need for an integrated, multi-modal diagnostic workflow.

## 5. Conclusions

SM-AHN is an aggressive hematologic malignancy whose diagnosis is frequently complicated by the overshadowing of neoplastic mast cells by the associated myeloid neoplasm. Accurate diagnosis hinges on a high index of suspicion and a comprehensive evaluation integrating morphology, immunohistochemistry, and molecular genetics. Identifying both components of this disease is critical for accurate prognostication and for guiding combined therapeutic strategies to improve patient outcomes.

## Figures and Tables

**Table 1:** Comprehensive clinicopathologic, cytogenetic, molecular, and treatment profile of the SM and SM-AHN patient cohort.

Diagnosis	Age (yrs)	Sex	Hb (g/dL)	Platelets (×10^3^/μL)	WBC (×10^3^/μL)	Serum Tryptase (ng/mL)	LDH (IU/L)	BM Cellularity (%)	Key BM Findings	Cytogenetics/FISH	NGS Findings	Treatment
SM + AML	35	M	7.1	20	79.29	85	1343	90	↑ blasts, eosinophilia, clusters > 15 mast cells (CD117+, CD25 dim+)	inv(16), +22; *CBFB* rearrangement (80%)	*KIT* D816Y (48%)	7 + 3 + Midostaurin
SM + CMML	47	M	8.3	18	18.97	139	320	↑ cellularity	monocytosis, dysgranulopoiesis, ↑ MCs (CD117+, CD25+)	+21 mosaicism	*KIT* D816V (64%), *ASXL1* (44%), *RUNX1* (87%)	Decitabine + Venetoclax + Midostaurin
SM + MDS-LB-SF3B1	93	F	12.2	234	7.42	71	ND	40	↓ erythropoiesis, large MC cluster (CD117+, CD25±)	Normal	*DNMT3A* (8%), *NRAS* (5%), *SF3B1*(18%,5%)	Luspatercept + Avapritinib
SM + MDS-EB1	66	M	7.4	475	5.67	55	ND	80–90	fibrosis (grade 2–3), spindle MCs, dyserythropoiesis, (CD117+, CD25+, CD2±)	del(9q)	*KIT* D816V (77%), *EZH2* (18%), *TET2* (33%)	Decitabine + Venetoclax + Midostaurin
SM + MDS-EB1	75	M	8.5	42	4.69	38	137	80–90	dyserythropoiesis, ↑ MCs	Normal	*KIT* D816V (25%), *U2AF1* (39%), *ASXL1* (41%), *CBL* (58%)	Decitabine + Venetoclax + Midostaurin
SM + MDS/MPN-RS-T	75	M	8.3	552	7.12	109	157	40–60	ring sideroblasts > 15%, multiple MC aggregates (CD117+, CD25+)	Y	*SF3B1* (40%), *DNMT3A* (42%), *U2AF1* (32%), *JAK2* V617F (5%). *KIT* D816V	Decitabine + Venetoclax + Midostaurin
Mast Cell Leukemia + MDS/MPN-U	74	F	6.9	58	4.68	409	402	60	fibrosis MF3, osteosclerosis, 20–30% MCs (CD117+, CD25-)	Normal	*ASXL1* (33%), *ETV6* (7%), *JAK2* (49%), *NRAS* (24%), *SRSF2* (46%), *TET2* (39%)	Decitabine + Venetoclax + Midostaurin
Isolated SM	69	F	14.7	308	7.61	62	132	30	Aggregates of spindled, neoplastic mast cells	Normal	*KIT* D816V (7%)	Avapritinib
Isolated SM	32	F	12.1	204	8.41	17	ND	70	,,	Normal	*KIT* D816V (19%)	Avapritinib
Isolated SM	76	F	12.4	187	5.82	178	ND	30–40	,,	Normal	*KIT* L576F (39%)	Avapritinib
Isolated SM	53	F	12.7	137	8.2	78	ND	50–60	,,	Normal	*KIT* D816V (22%)	Avapritinib
Isolated SM	55	M	13.8	435	6.54	91	ND	60–70	,,	Normal	*KIT* D816V (34%), *TET2* (3%)	Avapritinib
Isolated SM	53	F	14.1	341	6.06	47	174	50–60	,,	Normal	*KIT* D816V (26%), *TET2* (51%)	Avapritinib
Isolated SM	48	F	11.9	274	5.92	62	ND	40–50	,,	Normal	*KIT* D816V (19%), *DNMT3A* (4%), *EZH2* (2%)	Avapritinib
Isolated SM	55	F	15.1	168	5.36	29	191	60–70	,,	Normal	*KIT* D816V (15%), *DNMT3A* (4%)	Avapritinib

SM = Systemic mastocytosis, MDS = Myelodysplastic syndrome, MPN = Myeloproliferative neoplasm, CMML = Chronic myelomonocytic leukemia, AML = Acute myeloid leukemia, ↑ = Increased, ↓ = Decreased, ,, = Same as above.

## Data Availability

All data and information concerning this study will be made available from the corresponding authors upon reasonable request.

## Abbreviations

The following abbreviations are used in this manuscript:

SM	Systemic Mastocytosis
SM-AHN	Systemic Mastocytosis with associated hematologic neoplasm
MCL	Mast cell leukemia
MDS	Myelodysplastic syndrome/neoplasm
CMML	Chronic myelomonocytic leukemia
AML	Acute myeloid leukemia
NGS	Next generation sequencing
